# Genome Assembly of *Serratia marcescens* Type Strain ATCC 13880

**DOI:** 10.1128/genomeA.00967-14

**Published:** 2014-09-25

**Authors:** H. E. Daligault, K. W. Davenport, T. D. Minogue, S. M. Broomall, D. C. Bruce, P. S. Chain, S. R. Coyne, H. S. Gibbons, J. Jaissle, C. N. Rosenzweig, M. Scholz, H. Teshima, S. L. Johnson

**Affiliations:** a Diagnostic Systems Division (DSD), United States Army Medical Research Institute of Infectious Diseases (USAMRIID), Fort Detrick, Maryland, USA; b Los Alamos National Laboratory, Los Alamos, New Mexico, USA; c United States Army Edgewood Chemical Biological Center (ECBC), Aberdeen Proving Ground, Maryland, USA

## Abstract

*Serratia marcescens* ATCC 13880 is the type strain of the species and a commonly used quality control strain. Here, we present the annotated genome assembly of 5.13 Mbp (59.8% G+C content) as submitted to NCBI under accession no. JOVM00000000.

## GENOME ANNOUNCEMENT

We sequenced and assembled the genome of *Serratia marcescens* ATCC 13880 (CDC 81360, NCTC 10211) the type strain of the species originally isolated from pond water. The species has the ability to infect plants, invertebrates, and vertebrates (1). Human infection, generally found in children and the immunocompromised, may include a variety of organ systems and is often resistant to common antibiotics (2, 3). The type strain sequenced here is used in many research and diagnostic applications as a control strain.

High-quality genomic DNA was extracted from purified isolates of each strain using a QIAgen Genome Tip-500 at USARMIID-Diagnostic Systems Division (DSD). Specifically, 100-mL bacterial cultures were grown to stationary phase and nucleic acid extracted as per manufacturer’s recommendations. Draft sequence data included both high-coverage (302×) short-insert (300 ± 70-bp) and low coverage (5×) long-insert (7,404 ± 2,261-bp) Illumina datasets sequenced on the HiSeq 2000. The two libraries were assembled together in Newbler (Roche, version 2.6) and consensus sequences were computationally shredded into 2-kbp overlapping fake reads (shreds). The raw reads were also assembled in Velvet (version 1.2.08) and those consensus sequences were computationally shredded into 1.5-kbp overlapping shreds (4). Draft data from all platforms were then assembled together with Allpaths (version 44837) and the consensus sequences were computationally shredded into 10-kbp overlapping shreds (5). We then integrated the Newbler consensus shreds, Velvet consensus shreds, Allpaths consensus shreds, and a subset of the long-insert read-pairs using parallel Phrap (High Performance Software, version SPS-4.24). Possible misassemblies were corrected and some gap closure accomplished with manual editing in Consed (6–8).

Automatic annotation of the assembled *S. marcescens* 813-60 genome (4 contigs placed into 2 scaffolds) utilized an Ergatis based workflow at with minor manual curation. The final genome assembly is available in NCBI as accession no. JOVM00000000 and the raw data can be provided up on request. The total assembly is 5,131,448 bp long, contains 59.8% G+C content, 4,724 coding sequences, 22 rRNA sequences, and 99 tRNA sequences.

### Nucleotide sequence accession number.

The annotated genome assembly of *S. marcescens* 813-60 is available in GenBank under accession no. JOVM00000000.
